# Sea stack plots: Replacing bar charts with histograms

**DOI:** 10.1002/ece3.11237

**Published:** 2024-04-16

**Authors:** Alice Dorothy Stuart, Maja Ilić, Benno I. Simmons, William J. Sutherland

**Affiliations:** ^1^ School of Environmental Sciences University of East Anglia, Norwich Research Park Norwich UK; ^2^ Queen's University Belfast Belfast UK; ^3^ Centre for Ecology and Conservation, College of Life and Environmental Sciences University of Exeter, Cornwall Campus Penryn UK; ^4^ Department of Zoology University of Cambridge Cambridge UK

**Keywords:** bar charts, data distribution, data visualisation, histograms, summary statistics

## Abstract

Graphs in research articles can increase the comprehension of statistical data but may mislead readers if poorly designed. We propose a new plot type, the sea stack plot, which combines vertical histograms and summary statistics to represent large univariate datasets accurately, usefully, and efficiently. We compare five commonly used plot types (dot and whisker plots, boxplots, density plots, univariate scatter plots, and dot plots) to assess their relative strengths and weaknesses when representing distributions of data commonly observed in biological studies. We find the assessed plot types are either difficult to read at large sample sizes or have the potential to misrepresent certain distributions of data, showing the need for an improved method of data visualisation. We present an analysis of the plot types used in four ecology and conservation journals covering multiple areas of these research fields, finding widespread use of uninformative bar charts and dot and whisker plots (60% of all panels showing univariate data from multiple groups for the purpose of comparison). Some articles presented more informative figures by combining plot types (16% of panels), generally boxplots and a second layer such as a flat density plot, to better display the data. This shows an appetite for more effective plot types within conservation and ecology, which may further increase if accurate and user‐friendly plot types were made available. Finally, we describe sea stack plots and explain how they overcome the weaknesses associated with other alternatives to uninformative plots when used for large and/or unevenly distributed data. We provide a tool to create sea stack plots with our R package ‘seastackplot’, available through GitHub.

## Graphical abstract

Plots are an essential tool in the interpretation of published results but can be incomplete or misleading representations of the underlying data. We find that plot use in four journals spanning ecology, conservation, and behaviour relies heavily on bar charts and dot and whisker plots, which do not show data distribution. We put forwards sea stack plots as a novel plot type that overcomes many of the disadvantages of commonly used plots.

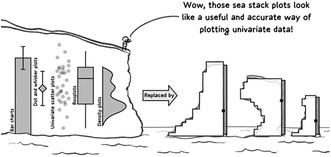

## 1 WHY GRAPH CHOICE IS IMPORTANT

Humans like to process quantitative information in graphic form (Pinker, 2014). This tendency is so pronounced that, for many, figures are the most important element when reading a scientific paper (Hubbard & Dunbar, 2017), with some looking at figures and tables before reading any of the text in the results section (Carey et al., 2020). Well‐designed graphs can significantly increase comprehension of statistical data (Feliciano et al., 1963) but, if used incorrectly, graphs can be highly misleading; this may be intentional (Huff, 2023; Wainer, 1984) or due to ineffective presentation of information (Kosslyn, 1989; Weissgerber et al., 2015, 2019). As such, it follows that graphs should be easily comprehended and clearly represent the data so as not to mislead researchers relying on them for the interpretation of results. For more information on the principles that make a good data visualisation, see Box 1.

Box 1 **Principles of good data visualisation.**

Although there have been many disparate attempts to create a theoretical framework for good data visualisation, a good graph can be defined as one with the following three properties (following Cleveland & McGill, 1985; Tufte, 2007; Zhu, 2007):

1. *Accuracy*: The attributes and structure of the graph should match those of the data, allowing the reader to form a judgement of the information encoded as close as possible to the ‘correct’ value. The graph should not be misleading.
2. *Utility*: The graph should help reader carry out the desired task, for example, understanding and comparing the distributions of data. The graph should effectively reveal the meaning and complexity of the data in a way that is closely integrated with any verbal or statistical descriptions.
3. *Efficiency*: The graph should be easy to work out how to read and allow the desired task to be completed quickly and easily. The graph should have a high data density and not contain unnecessary information, features, or blank space.

**FIGURE 1:**
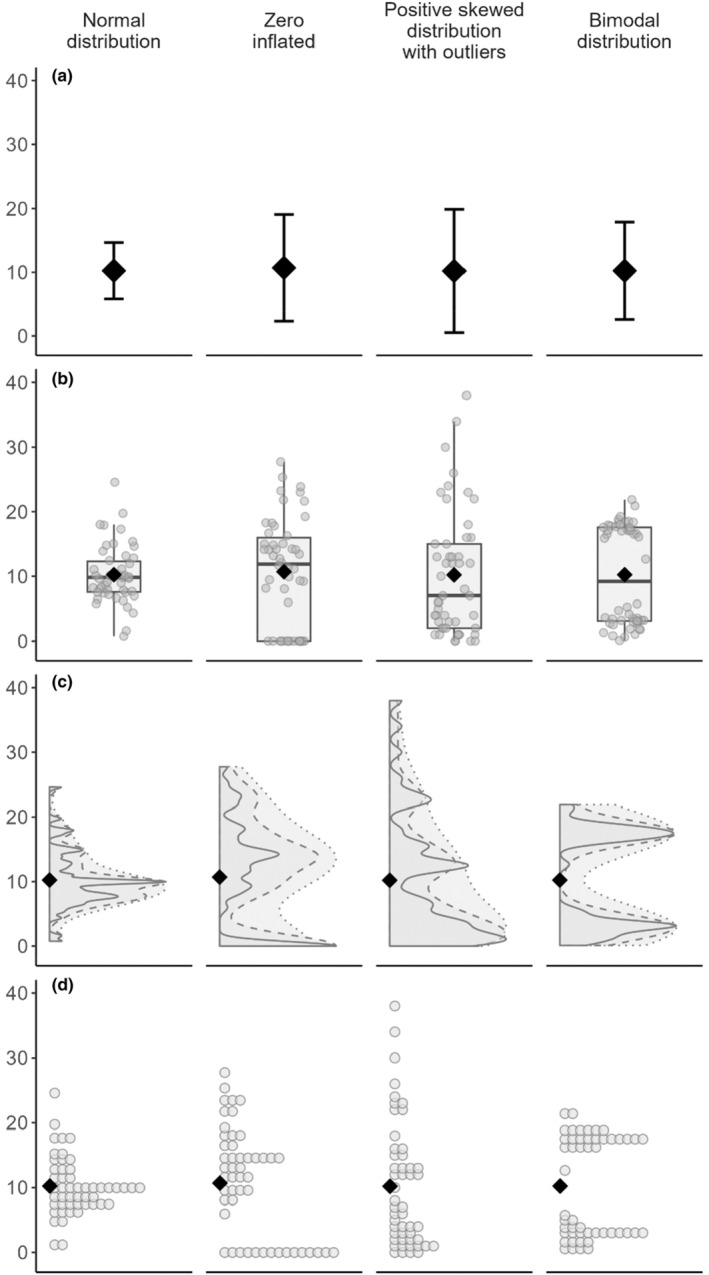
Comparison of different plotting options to represent the distribution of the data. Panels from the top row to the bottom row: (a) simple dot and whisker plot with mean and error bar (± 1 SD); (b) boxplots with univariate scatter plot; (c) flat density plots with different degrees of smoothness (solid line: adjust = 0.2; dashed line: adjust = 0.5; dotted line: adjust = 1); (d) dot plots. These series of plots are based on four data groups representing common data distributions in biological studies (left to right): normal distribution; zero inflated; positive skewed distribution with outliers; bimodal distribution. Each group was simulated with the same sample size (*n* = 50) and intended mean value (10), shown with a black diamond. Code used to create the dummy data and the plots can be found in Appendix A.

Considerable effort has been expended to develop new visualisation tools and propose modern, attractive, and informative plots, with different levels of complexity suitable for a range of data and research questions. The plots in Figure 1 show a set of graph types frequently used to represent four different distributions commonly observed in biological studies: normal, zero inflated, positively skewed with outliers, and bimodal. The plots are arranged from the most aggregated, a dot and whisker plot, at the top; to the least aggregated, a binned dot plot, at the bottom.

The dot and whisker plot (Figure 1a) and bar charts show only mean (dot/bar) and dispersion, generally standard deviation, (whisker/error bars). This allows the reader to draw conclusions about the dispersion of the data, but not to make any assertions about the distribution of the underlying data. Such plots provide the same amount of information as when bar charts are used for continuous data, as they generally show the same statistics. Much discussion has been had about the inadequacy of bar charts for scientific data visualisation, with Weissgerber et al. (2015, 2019) representing a good introduction.

Boxplots (Figure 1b) show more summary statistics, generally including the median, quartiles, and extreme values (beyond 1.5 × interquartile range). This allows for the reader to draw conclusions about the distribution of the data, for example, if the distribution is skewed or if there is a large tail on one or both ends. When used alone, they still lack some important information, for example, one cannot tell if the underlying data is bimodal or has other unusual properties. For small sample sizes, this can be solved for some datasets by adding the raw data as a univariate scatterplot, as shown in Figure 1b. However, as is discussed below, this solution does not suit larger sample sizes.

Density plots (Figure 1c) are often touted as being an effective alternative to boxplots. These are attractive in their design, showing a smoothed density curve of the observations along the data points (Hintze & Nelson, 1998). Smoothed density plots (Figure 1c; dotted outer line), however, do not always accurately represent the data; for example, the shape of the smoothed density plot spuriously suggests that there are datapoints present between 0 and 5, which is not the case (see boxplot or dot plots representing the same data distribution). Further, depending on the software used and chosen settings, the smoothing can make some distributions of data that appear to have a spuriously large sample size: for example, the smoothed density plot representing zero inflated data in Figure 1c has a considerably larger area than that showing normally distributed data, despite both groups having the same sample size (*n* = 50). These features of the plot can be controlled for, if the user is familiar with the default settings, and should be additionally reported in the published literature. Unsmoothed flat density plots (Figure 1c; solid outer line) are much more accurate. However, these are less attractive than the other plotting options and can be hard to interpret visually when there are many small ‘spikes’ close together, such as in Figure 1c normal distribution unsmoothed density plot.

Univariate scatter plots (shown overlayed on the boxplot in Figure 1b) show data truthfully, albeit often with jitter to improve interpretation where there are overlapping points, but can be difficult to read and interpret with anything but very small datasets (≤ 20 points) (Weissgerber et al., 2019). For large datasets, especially when many points are overlapping, it becomes difficult to visually inspect the underlying distribution as this requires interpretation of point density, which humans do not perceive particularly accurately (Cleveland & McGill, 1985). This issue is particularly prevalent when the datapoints are relatively close together, as in the normal distribution in Figure 1b. One way to improve this is to stack or arrange the dots into dot plots (Figure 1d). These show the data relatively accurately and work well with small sample sizes. However, with increasing sample sizes, fitting all dots into the panel becomes difficult; this may be avoided by forcing the dots to overlap, which reduces the overall width of the dot plot. Where sample sizes are very large, ensuring individual dots are visible requires further fixes like increased dot transparency, at which point the plot becomes less clear, defeating the original purpose of simply using raw data points. It is also of note that sometimes dot plots are plotted using simple histogram binning, which may not be immediately clear to the interpreter and reduces the accuracy of the plot (Wilkinson, 1999). Further, some variants, such as beeswarm plots, create visual artefacts that hinder interpretation (Galili, 2016) and therefore should be avoided.

One way to overcome the limitations of the above‐mentioned individual plot types is to combine them, thus allowing the information from two or more plots to be read within one figure. Density plots can be plotted with a boxplot to show summary statistics (Hintze & Nelson, 1998). More modern examples of combined plots exist such as ‘raincloud plots’ (Allen et al., 2019), which are a combination of a horizontal, flat density plot, and the raw data, usually plotted as jittered points to allow data points with equal/similar values to be visible. Combined plots, however, will carry through some of the limitations of the plot types they are made up of; an example of this would be using raincloud plots for a very large dataset, where the density plot remains prone to misrepresenting the data if incorrect settings are used, while the univariate scatterplot would provide little information due to overplotting.

## 2 WHAT PLOT TYPES ARE PAPERS IN ECOLOGY AND CONSERVATION USING?

**FIGURE 2:**
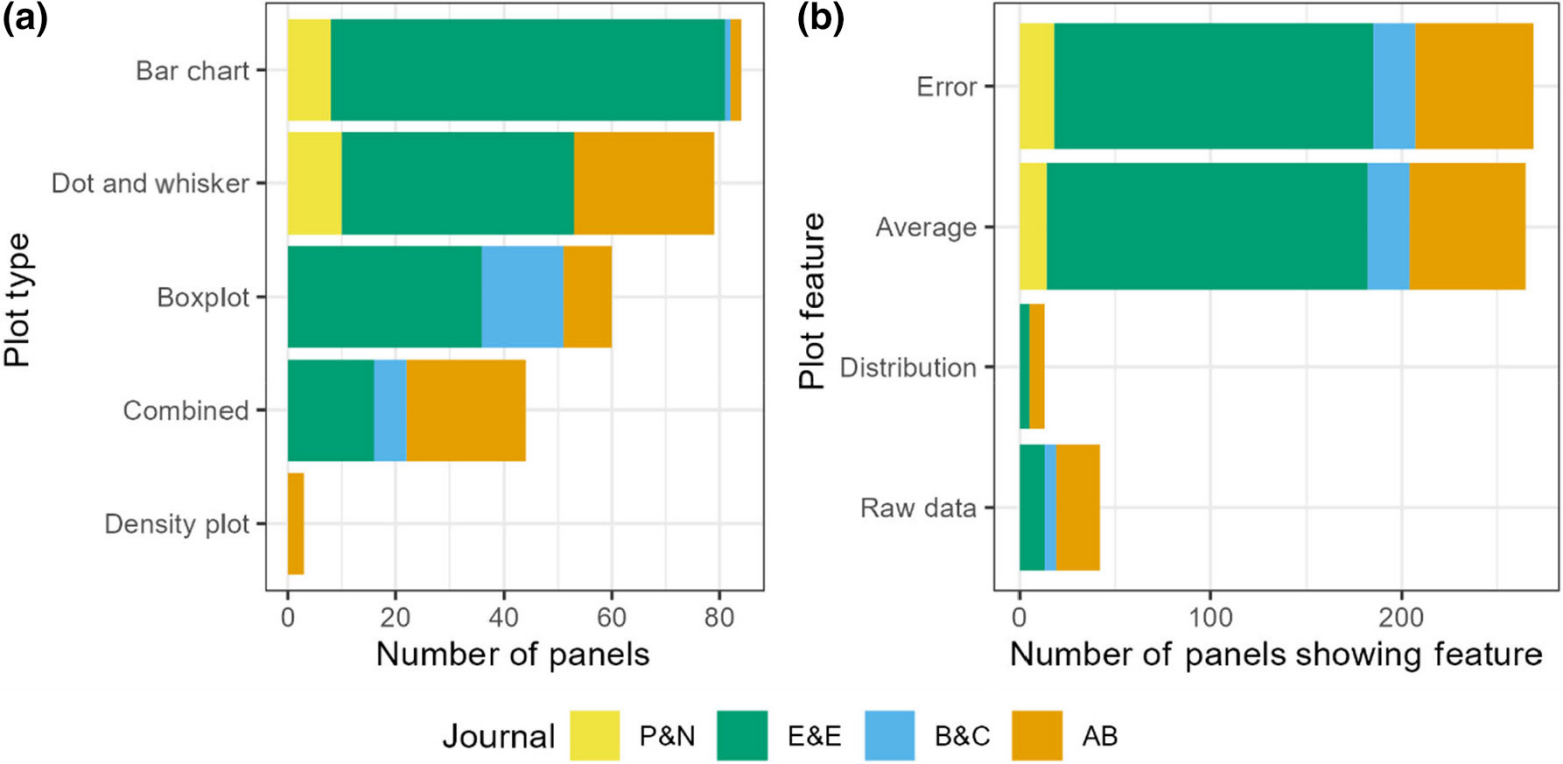
Count data of plot types and features in the 270 figure panels used to represent continuous univariate data in four ecology and conservation journals (*P&N: People and Nature*; *E&E: Ecology and Evolution*; *B&C: Biodiversity & Conservation*; and *AB: Animal Behaviour*) shows prevalent use of highly aggregated plot types in ecology with associated failure to show distribution or raw data in most figure panels.

Given the failings of bar graphs, and their related dot and whisker plots, it is worrying that they remain prevalent in the reporting of results in many fields (Riedel et al., 2022; Weissgerber et al., 2015, 2019), including ecology (Friedman, 2021). Multiple calls have been made to phase out the use of bar graphs (Weissgerber et al., 2015, 2019) leaving potential for the state of graph use in ecology to have improved since the articles analysed by Friedman (*Journal of Ecology*, 1996–2016) were published. To assess whether ecology and conservation research articles still suffer from poor graph use, we analysed the graph types used to plot group comparisons of continuous univariate data (e.g., size measured across multiple species) in the most recently completed (at the time of analysis) issues of four journals (*People and Nature*, *Ecology and Evolution*, *Biodiversity and Conservation*, and *Animal Behaviour*). This was not intended to be a systematic review, instead these journals were chosen as they cover a range of topics within ecology, conservation, and behaviour with a diversity of publishers (the British Ecological Society via Wiley, Wiley, Springer, and Elsevier, respectively) thus would be broad enough to assess whether poor graph use persists. All plots analysed fell into one of the following categories: bar chart, dot and whisker, boxplot, density plot, or combined (two or more plot types shown together, for example, a boxplot shown with a univariate scatterplot). More details of the methods used to gather these data can be found in Appendix B. Of 1007 figure panels within the 92 peer‐reviewed research articles analysed, 270 showed continuous univariate data, a breakdown of these by plot type is shown in Figure 2.

Similar to the results of Weissgerber et al. (2015) for physiology, Weissgerber et al. (2019) for peripheral vascular disease, and Riedel et al. (2022) for papers in PubMed, research articles in ecology, behaviour and conservation journals still predominantly rely on highly aggregated types of plots (Figure 2a). Together bar charts (84 panels, 31%) and dot and whisker plots (79 panels, 29%) were more common than all other plot types combined, with boxplots being the next most common plot type (60 panels, 22%). This was worryingly reflected in the low proportion of plots showing either (or both) the underlying distribution or raw data (Figure 2b; 50 of the 270 panels, 19%).

The more informative panels tended to use multiple plot types combined into a single figure panel to show more information (43 panels, 16%), as discussed above. A breakdown of the combined plots is shown in Table 1. Most combined plots used a boxplot as a base, with a second layer showing the distribution of the data (40/43 combined plot panels). The most common secondary plot was univariate scatterplots (28/43 panels), which, as discussed above, becomes difficult to read at high sample sizes. The use of combined plots in the research articles analysed shows there is a need within conservation and ecology for more effective plot types, which may further increase if an accurate and user‐friendly plot types were made available.

**TABLE 1:** A breakdown of the plot combinations used in combined plots (*n* = 43 panels).

Figure type	Panels	Featured in
Boxplot and univariate scatterplot	28	Brogi et al. (2022); Driscoll et al. (2022); Farji‐Brener et al. (2022); Hays et al. (2022); Hills & Webster (2022); Houdelier et al. (2022); Gibert et al. (2023); Keppner et al. (2023); and Pilakouta et al. (2023)
Boxplot and stacked dot	6	Geldenhuys et al. (2022)
Boxplot and histogram	4	Mason et al. (2023)
Dot and whisker and stacked dot	3	Ratz et al. (2022)
Density and boxplot	2	Lymbery et al. (2022) and Marinček et al. (2023)
Density and dotplot	1	Romano et al. (2022)

## 3 THE SOLUTION—SEA STACK PLOTS

**FIGURE 3:**
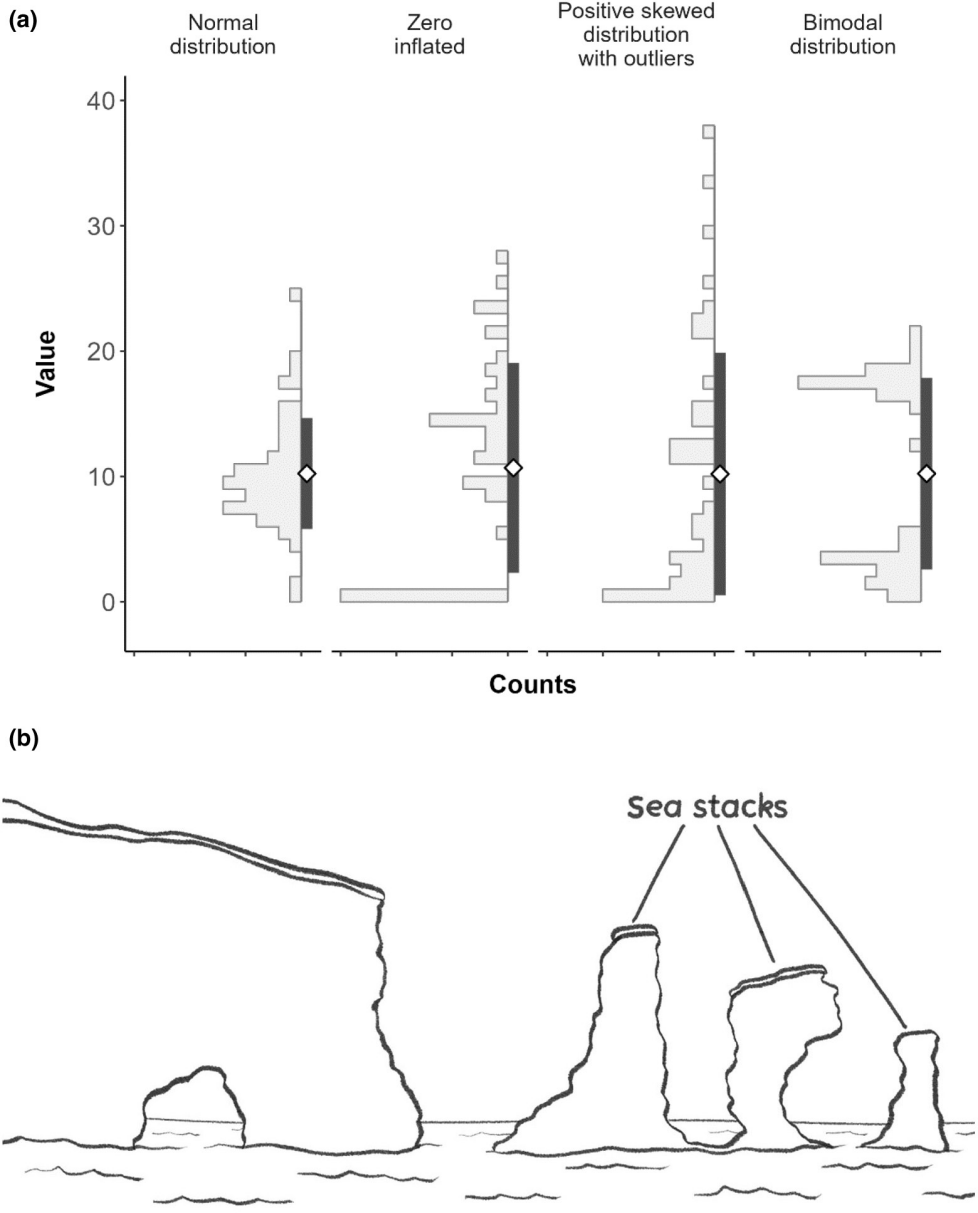
(a) Stack plots: a combination of histograms or ridges and summary statistics. The white diamond represents the mean, while the vertical dark grey bar represents the standard deviation (mean ± 1 SD). Similarly, non‐parametric summary statistics (such as the median or the quantile ranges) can be added to the plot (see Figure C1). The actual data values (measures) are given on the *y*‐axis, and counts are represented as ticks on the *x*‐axis, the minimum value of the counts will always be 0 and the maximum value can be provided in the figure legend (here 15). (b) An illustration of sea stacks, the namesake of sea stack plots.

Here we present the sea stack plot, which combines a vertical histogram and summary statistics (Figure 3a). The plots were named for their resemblance to sea stacks (Figure 3b), such as the Old Man of Hoy in Orkney, UK. Histograms are a robust and effective method to show distribution: where the bin width is chosen properly, the data is represented in biologically meaningful bins, and all data points are equally treated and plotted, both within and across data groups. The bin width depends on the data and the hypothesis, for example, if the histogram should be used to represent size distributions, as it is often the case in fisheries management studies (length–frequency histograms, see figure 2 in Brenden et al., 2007), bin width of 1 unit would be appropriate, for example, 1 cm. This also allows for a direct estimation and representation of actual counts in each bin and for an easy comparison between group sizes.

We believe the combination of histogram with relevant summary statistics (such as mean and confidence interval) results in a highly useful graph, providing more information than a simple histogram but remaining easily interpretable. Further, sea stack plots solve many of the problems seen in the plot types discussed in Section 2. The histograms used to show distribution retain consistently high accuracy across medium and large sample sizes, unlike univariate scatter plots, and, unlike density plots (and their use in violin and raincloud plots), they are not prone to over‐ or under‐represent certain aspects or distributions of data (such as zero inflation). Moreover, they retain high efficiency when plotting very large sample sizes as no space is taken up by an overplotted univariate scatterplot providing little extra information.

**FIGURE C1:**
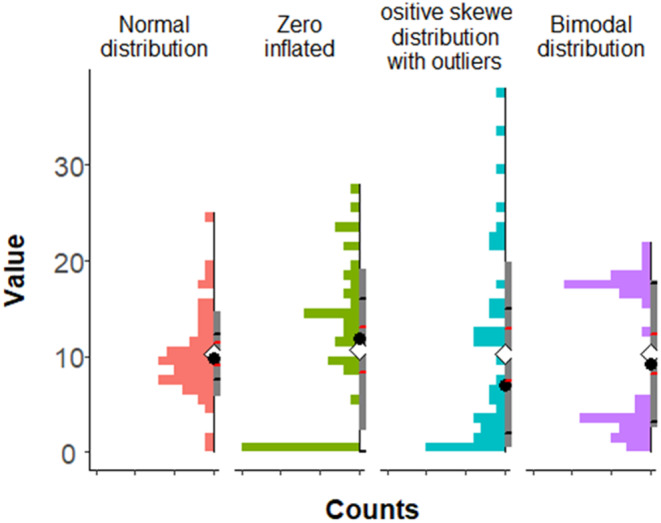
Sea stack plots with both parametric (mean, standard deviation, and 95% confidence interval) and non‐parametric (median, and 50% quantiles) summary statistics. As in Figure 3, the white diamond represents the mean, while the vertical dark grey bar represents the standard deviation (mean ± 1 SD). Additionally, the black circle represents the median; the horizontal red lines (perpendicular to the grey error bar) represent the 95% confidence interval (mean ± 1.96 * standard error of the mean); and the horizontal black lines represent the 50% quantiles (median ± 25% of the data). The actual data values (measures) are given on the y‐axis, and counts are represented as ticks on the x‐axis, while the range of the counts (first and last tick) can be provided in the figure legend (here: [0,15]).

Although we chose the vertical direction over the horizontal direction, the plots can easily be produced as horizontal sea stack plots if necessary. A range of summary statistics can be shown depending on what is most appropriate for the data (for recommendations on representing error in ecological datasets, see Greenacre, 2016): we here chose to show the mean (white diamond) and the interval mean ± standard deviation (SD), which is represented as the vertical dark grey bar. Alternatively, or even additionally, confidence intervals and non‐parametric summary statistics, such as the median and the quantile ranges, can be plotted (see Figure C1 in Appendix C). We have specifically chosen to show the histograms on the left‐hand side so that, when the printed paper or the PDF is flipped to the right, the y‐axis is on the top and can still be read from left (smaller values) to right (larger values). We believe this to be more intuitive compared to all other plots, which usually have the opposite direction.

There is considerable scope for sea stack plots to improve the presentation of univariate data within ecological literature; in the papers analysed, almost all of the 270 panels showing continuous univariate data (27% of all panels) could be replaced with a sea stack plot to ensure a more transparent and informative presentation of the data. We recommend using sea stack plots for continuous data with sample size *n* ≧ 20. Sea stack plots have limited use for smaller sample sizes—in such cases, with *n* < 20, in which cases we recommend showing a boxplot or simple mean with an error bar in combination with a univariate scatter plot of the raw data will be readable and the most accurate and transparent way of portraying the data (Weissgerber et al., 2015). Further, we do not recommend sea stack plots for paired data where observations in one dataset have a meaningful and one‐to‐one relationship with those in another, for example, duplicate measurements, as in this case it is necessary to show raw data to allow for direct comparison.

To make this plot more accessible we have created seastackplot, a user‐friendly R package available on GitHub. The package can be downloaded from the repository https://github.com/Al‐Stu/seastackplot using the install_github() function in the devtools R package (Wickham et al., 2022) with the command: devtools::install_github(‘Al‐Stu/seastackplot’). A full explanation of how to use the package is available in Appendix D.

## 4 CONCLUSION

Good communication of science relies on the use of accurate, useful and efficient graphs to present quantitative findings. We believe that sea stack plots are part of the solution to tackling the continued use of poor graphing methods for continuous univariate data within ecology and have discussed their merits relative to commonly used graph types in this article. We echo previous papers' calls for journals to be stricter on the methods used to plot continuous data and hope the seastackplot R package and explanation of how to use it within this article allow researchers in ecology and beyond to take sea stack plots forwards to improve transparency and consistency regarding data visualisation in research articles.

## AUTHOR CONTRIBUTIONS

**Alice Dorothy Stuart:** Formal analysis (lead); investigation (lead); methodology (equal); software (equal); visualization (equal); writing – review and editing (equal). **Maja Ilić:** Conceptualization (equal); software (equal); visualization (equal); writing – original draft (equal); writing – review and editing (equal). **Benno I. Simmons:** Conceptualization (equal); writing – review and editing (equal). **William J. Sutherland:** Conceptualization (equal); writing – review and editing (equal).

## CONFLICT OF INTEREST STATEMENT

The authors have no competing interests to declare.

## Supporting information


Appendix S1.

## Data Availability

All codes that are used to create dummy data for plots are included in Appendix A. The code used to create the plots in this article and data on plot use are available as Supporting Information alongside the article.

## APPENDIX A Creating dummy data.

To compare the suitability of different plot types to accurately present data (including the summary statistics and distribution), we created a dummy dataset consisting of four subsets, each with the sample size *n* = 50 and intended mean of 10, representing four frequently observed data distributions in biological studies: normal, zero inflated, positive skewed distribution with outliers, and bimodal distribution. These data were generated in R (version 4.2.2) using the following code:

To avoid any negative values (as they are biologically not possible, e.g., a measure of size can never be below 0), we used absolute values of all generated data.

df$Value <‐ abs(df$Value)

## APPENDIX B Analysis of existing plot use.

To assess the plots used to represent continuous univariate data, we analysed research articles in the most recent complete issues (at the time of analysis) of *Animal Behaviour* (Volume 194, December 2022; 24 articles), *People and Nature* (Volume 4, Issue 6, December 2022; 20 articles of which 15 were research articles), *Biodiversity and Conservation* (Volume 31, Issue 13–14, December 2022; 11 articles of which 9 were original papers), and *Ecology and Evolution* (Volume 13, Issue 1, January 2023; 47 articles of which 44 were research articles); the number of articles from each journal is also shown in Table B1. The journals were chosen because they cover a range of topics within ecology and conservation and are issued by a variety of publishers (Elsevier, the British Ecological Society via Wiley, Springer, and Wiley respectively).

**TABLE B1:** Number of articles analysed from each journal.

Journal	Article type	No of articles
Animal Behaviour	Research article	24
Biodiversity & Conservation	Original paper	9
	Other	2
Ecology & Evolution	Research article	44
	Other	3
People & Nature	Research article	15
	Other	

The main body of each paper was read online to find any figures. To account for the use of multiple plot or diagram types in a single figure, where a figure had multiple panels (e.g. Figure 1a,b), each was coded individually. A total of 1007 figure panels were assessed. Each panel was coded for the section it occurred in and type of data shown. Where figure panels were non‐graphical, or showed data types that could not be reproduced in a sea stack plot (e.g., continuous‐continuous data like that shown in a scatterplot) they were coded as being the incorrect data type and not taken forwards for further coding (737 panels). The remaining 270 panels were coded by type and whether they showed an average, error (counted as standard deviation, standard error of the mean, interquartile range, or range), the distribution of the data, and/or the raw data themselves. For analysis, similar plot types were combined for clarity (see Table B2). The number of plots of each type is shown in Table B3 and the occurrence of different plot features in Table B4.

**TABLE B2:** Codes and final plot types used for analysis.

Plot type	Plot codes
Boxplot	box
	notched_box
Dot and whisker	dot_and_whisker
Bar chart	Bar
Combined	dot_and_boxplot
	stacked_dot^a^
	violin_and_boxplot
	dot_and_violin
	box_and_histogram
	boxplot_stacked_dot
Density plot	violin

^a^ All stacked dot plots also had dot and whisker.

**TABLE B3:** Number of panels of each plot types within the 270 panels showing the correct data type.

Plot type	Panels
Bar chart	84
Dot and whisker	79
Boxplot	60
Combined	44
Density	3

**TABLE B4:** Number of panels showing each plot feature within the 270 panels showing the correct data type.

Plot feature	Panels
Error	269
Average	265
Raw data	42
Distribution	13

## APPENDIX D Seastackplot tutorial.

### Install packages

As the package is available through GitHub, the remotes package is required to install it. The seastackplot also relies on the package ggplot2, so we need to install this as well. We then need to load the seastackplot package to use the functions.

### Generate data to plot

This demonstration will use the same generated data as plotted in the paper to show differences between other graph types.

### Plot using sea_stack_plot() function

The easiest way to plot a sea stack plot is using the sea_stack_plot() function. To create a sea stack plot using the sea_stack_plot(), you need to make sure your data is long format (see here for an explanation of long vs wide format data http://www.cookbook‐r.com/Manipulating_data/Converting_data_between_wide_and_long_format/).

To plot a default sea stack plot with mean and standard deviation, the sea_stack_plot() function required the following variables: *data your long format data frame or tibble *data.column the name of the column your values are in * group.column the name of the column your group names are in

The sea_stack_plot() function also includes the following variables for plot customisation: *data.label the desired title for the data axis, if left NULL (default), will be the value of data. column

- bin.width the bin width being used to plot
- mean.size the size of the diamond showing the mean
- median.size the size of the point showing the median
- orientation “vertical” or “horizontal”, whether or not the plot is being plotted vertically (default) or horizontally
- show.mean whether the mean should be plotted
- show.median whether the median should be plotted
- show.standard.dev whether the standard deviation rectangle should be plotted
- show.confidence.int whether the confidence interval should be plotted
- show.quantiles whether quantiles should be plotted
- ci.line.length how long the ticks representing the confidence intervals should be
- quant.line.length how long the ticks representing the quantiles should be
